# Dental caries in permanent first molars: implications for health surveillance

**DOI:** 10.1590/1980-549720260026.supl.1

**Published:** 2026-07-31

**Authors:** Paulo Capel Narvai, Antonio Carlos Frias, Carlos Cesar da Silva Soares, Jose Leopoldo Ferreira Antunes

**Affiliations:** I Universidade de São Paulo, School of Public Health, Department of Policy, Management and Health – São Paulo (SP), Brazil.; II Universidade de São Paulo, School of Dentistry, Department of Social Dentistry – São Paulo (SP), Brazil.; III Universidade de São Paulo, School of Public Health, Department of Epidemiology – São Paulo (SP), Brazil.

**Keywords:** DMF index, Dental caries susceptibility, Water fluoridation, Preventive health services, Public health surveillance

## Abstract

**Objective:**

To analyze the participation of the first permanent molars (M1) in the composition of the DMFT index, with or without exposure to fluoridated water (FW), its potential implications for health surveillance.

**Methods:**

its potential implications for health surveillance FW. Mean DMFT values and the percentage contributions of different tooth groups to these values were calculated.

**Results:**

Mean DMFT values were 2.66, 2.49, and 2.21 for capitals without FW in 2003, 2010, and 2023, respectively, and 1.58, 1.36, and 0.96 for capitals with FW. Between 2003 and 2023, mean DMFT values declined by 16.2% in capitals without FW and by 39.2% in capitals with FW. In 2003, the contribution of M1 to the DMFT index was 70.51% among those exposed to FW and 63.61% among those not exposed. In 2010 and 2023, these contributions were 67.95% and 57.68%, and 63.61% and 49.23%, respectively.

**Conclusion:**

The controlled presence of fluoride in water contributed to an increased percentage contribution of M1 to the composition of the DMFT index, whereas this proportion decreased in the absence of this preventive measure. However, this effect was not observed for premolars and second molars. This variation is relevant to oral health surveillance strategies, particularly activities aimed at the active detection of early caries lesions and their treatment.

## INTRODUCTION

The first permanent molars (M1s) constitute the group of teeth most susceptible to dental caries. For this reason, their contribution to the DMFT index is well recognized among epidemiologists investigating the distribution of this disease in populations. In general, this proportion ranges, across all ages, from one-third to two-thirds of the index value, regardless of disease magnitude. In contrast, canines represent the group least affected by dental caries^1^. In the pioneering study by Klein, Palmer, and Knutson (1938), in which the DMFT index was first proposed, canines accounted for only 0.34% of the total index value among individuals aged 6 to 15 years (*n* = 4,416). In comparison, M1s contributed 77.59% of the total value, of which 51.43% corresponded to the mandibular molars^2^.

At the index age of 12 years, prevalence characteristics do not significantly influence the aforementioned proportion of M1 contribution to DMFT index values. It is assumed that this pattern of caries distribution arises from the morphological characteristics of the dental crowns across different tooth groups^3^.

In Birmingham, UK, where the public water supply has been fluoridated (fluoridated water – FW) since 1964, 40% of 11-year-old children had caries-free M1s in 1980. This proportion was compared with that observed among children of the same age in Wolverhampton, who had not been exposed to FW, among whom 78% presented caries in at least 1 of the 4 M1s. The number of first molars extracted in Wolverhampton was nearly four times higher than that recorded in Birmingham^4^. In Brazil, Lopes et al. evaluated, in 1985, the prevalence of caries in M1s among children who were born and had always resided in two communities in the state of Piauí: Teresina, with a fluoridated public water supply (0.68 mgF/L), and Barras, without FW. Although the contribution of M1s to the composition of the DMFT index was not analyzed, the authors concluded that exposure to community water fluoridation (Teresina) contributed to reducing the magnitude of disease manifestation across all dental surfaces, including M1s, compared with that observed in the absence of exposure (Barras)^5^.

Although, within the context of health planning and programming, the epidemiological characteristics of M1s among dental groups are of particular interest for the planning and organization of oral health surveillance actions, the consistent body of knowledge available on M1s during the second half of the 20th century — not only regarding health outcomes but also their importance for maintaining adequate dental occlusion — appeared to be considered sufficient by researchers, as the topic virtually disappeared from the literature on caries prevention. In this context, Rocca et al., in 1979, stated that “relatively few researchers have been concerned with evaluating data specifically related to first molars, considering their biological significance and high susceptibility to carious lesions”^6^.

An international exception was the study conducted by Linkosalo^7^, which analyzed differences in caries experience in first permanent molars by calculating the DMFT index specifically for M1s in two groups of Finnish children born in 1963 and living in cities with and without community water fluoridation. The study concluded that exposure to FW alone is insufficient to constitute an effective preventive strategy. However, during the first decade of the 21st century, Batchelor and Sheiham redirected attention to M1s and demonstrated that, comparatively, the occlusal surfaces of these teeth are the most susceptible to caries. The authors also reported that the occurrence of caries in the pits and fissures of the lower first molars is strongly associated with an increased risk of involvement of second molars and premolars^3^.

The prevalence, incidence, and progression rates of caries in permanent teeth follow universally recognized patterns that are considered by professionals responsible for health planning and programming. These patterns can be summarized as follows: a) caries levels follow trend lines, implying that, at a given age, they may exhibit predictive value regarding levels observed in subsequent age groups; b) at the population level, as the mean DMFT index increases, the proportion of caries-free individuals decreases, while disease dispersion within the population increases; c) a specific mathematical relationship exists between the mean DMFT index (in which the unit of measurement is the tooth) and the mean DMFT index (in which the unit of measurement is the dental surface); d) susceptibility to caries follows a hierarchy according to tooth type and dental surface, with well-defined patterns of occurrence; e) changes in mean DMFT values for individuals and population groups are not linear but scalable, with groups of teeth and dental surfaces exhibiting similar levels of “resistance” to caries; and f) as the mean DMFT index decreases, resistance to enamel demineralization processes increases during the post-eruptive maturation phase. Although improvements in population oral health conditions reduce disease progression, the hierarchy of susceptibility to dental caries remains unchanged, regardless of fluoride exposure^1^.

Although the safety and effectiveness of community water fluoridation in reducing the prevalence and magnitude of dental caries are supported by scientific evidence^8^, thereby benefiting all dental groups, differences are recognized in the extent to which this “preventive effect” is expressed among different groups of teeth.

Given that fluoride exposure generally reduces the contribution of incisors and canines to the DMFT index, a logical implication is that, among individuals exposed to fluoride, the proportional contribution of M1s to this composition increases. However, although the preventive efficacy of fluoride across all dental groups is well established, owing to the similarity of its mechanism of action on demineralization and remineralization processes in dental enamel, the magnitude of the difference in the proportional contribution of M1s to the DMFT index under conditions of fluoride exposure *versus* non-exposure remains unclear.

The theoretical premise underlying this article is that scientific evidence supports a positive association between exposure to fluoridated public water supplies and a lower prevalence of dental caries. However, within the context of oral health planning and programming, this association may lead to the assumption that, when exposure to FW occurs and its preventive effectiveness is acknowledged, the protective effect of the measure is proportionally equivalent across all dental groups. This article examines that assumption by evaluating the magnitude of the difference in the proportional contribution of M1s to the composition of the DMFT index under conditions of exposure and non-exposure to FW. The aim is to assess the extent to which exposure to FW modifies this proportion and to explore the potential implications for the planning of oral health surveillance actions.

## METHODS

Secondary data obtained from samples of national oral health surveys conducted by the Brazilian Ministry of Health through the SB Brasil Project in 2003 (n=5,243)^9^, 2010 (n=5,578)^10^, and 2023 (n=4,721)^11^ were analyzed. The analysis included 12-year-old individuals from Brazilian state capitals with or without exposure to FW^12^. Mean DMFT index values and the percentage contributions of different tooth types to these values were calculated. Sample elements from capitals with at least five consecutive years of water fluoridation were classified as exposed to FW^13,14^. Primary data were derived from population-based epidemiological surveys conducted using the methodology recommended by the World Health Organization (WHO) for assessing the prevalence and severity of dental caries^15^.

The decision to use data exclusively from state capitals, rather than from the total national population represented (available sample universe), was based on the limited availability of information regarding fluoride concentrations in public water supplies in Brazil^16^ during the study period^17^. According to the Water Quality Surveillance Information System for Human Consumption (*Sistema de Informação de Vigilância da Qualidade da Água para Consumo Humano* – Sisagua), 31% of municipalities have insufficient records. This heterogeneity compromised the feasibility of conducting analyses using nationwide data. Chart 1 presents the lists of state capitals with and without FW in 2003, 2010, and 2023. The source triangulation technique was employed to validate the information.

**Chart 1:** List of Brazilian state capitals with and without fluoridated water in 2003 and 2010, and in 2023.

Year	Fluoridated water	
	Yes (year of implementation)	No
2003 and 2010	Aracaju (1985) Belo Horizonte (1977) Brasília (1968) Campo Grande (1985) Curitiba (1958) Florianópolis (1982) Fortaleza (1989) Goiânia (1985) Porto Alegre (1975) Rio de Janeiro (1985) * Salvador (1996) São Paulo (1985) Teresina (1997) Vitória (1982)	Belém Boa Vista Cuiabá João Pessoa Macapá Maceió Manaus Natal Palmas Porto Velho Recife Rio Branco São Luís
2023	In addition to the capitals with FW in 2003 and 2010, Cuiabá and Palmas were added in 2023. Palmas initiated fluoridation in 2016 and Cuiabá in 2017.	Capitals without FW in 2003 and 2010 remained in this category in 2023, except for Cuiabá and Palmas.

FW: fluoridated water; *information could not be confirmed through triangulation.

Sources: Database from the SB Brasil 2003 Project; Database from the SB Brasil 2010 Project; Souza et al.^18^; Barros et al.^19^; Freire et al.^20^; Cardoso et al.^21^; Bleicher et al.^22^; Cesa et al.^23^; Silva et al.^24^; Gonçalves et al.^25^; Narvai et al.^12^; Peres et al.^26^.

The hypothesis of equality in the proportional contribution of different tooth types to the composition of the DMFT index, under conditions of exposure and non-exposure to FW, was tested for the years 2003, 2010, and 2023 using the z-test, with a significance level of 5%^27^. Hypothesis testing was performed separately for each tooth group.

For comparative analysis of mean DMFT values between capitals with and without FW, a *t*-test was performed, adopting a significance level of 5%^27^. However, it is important to emphasize that the objective of this article is not to conduct an epidemiological analysis of dental caries in capitals with and without FW. For this reason, DMFT values are presented as originally reported following completion of the surveys conducted by the Ministry of Health. Accordingly, it is reiterated that the objective of this study is exclusively focused on patterns of disease distribution among different dental groups in capitals with and without FW, as represented in the samples obtained from the epidemiological surveys on which these data are based.

The aforementioned population surveys employed complex sampling designs. Therefore, considering the purpose of the present study, the analyses conducted were restricted to the respective samples. This clarification regarding complex sampling designs is necessary because it represents a central aspect of the study conception and methodological approach. Accordingly, the statistical analyses do not refer to the “full” DMFT values of the units of analysis (state capitals) at 12 years of age, but exclusively to M1s, since the objective of this study was neither to infer population parameters nor to analyze the prevalence of dental caries in the capitals as estimated by the overall DMFT index values. By focusing specifically on M1s, in accordance with the study objective, the “full” DMFT values were calculated, and the proportions representing the contribution of M1s to these respective values were subsequently determined.

### Data Availability Statement

The entire dataset supporting the results of this study is publicly available upon request to the Ministry of Health via email: cosab@saude.gov.br.

## RESULTS

**Figure 1:**
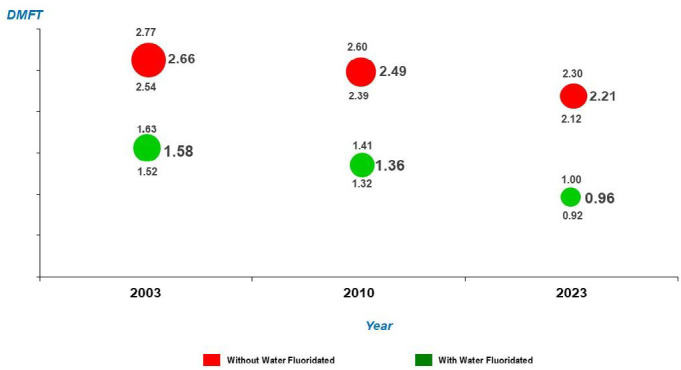
Mean DMFT index values at 12 years of age in Brazilian capitals with and without fluoridated water, in 2003 (n=5,243), 2010 (n=5,578), and 2023 (n=4,721).

Note: The size of the circle around the point estimate of the mean indicates the (95%) confidence interval estimate of the population parameter.

Source: Brazilian Ministry of Health. SB Brasil Projects 2003, 2010, and 2023.

Figure 1 presents the mean values and corresponding (95%) confidence intervals for the DMFT index in capitals with and without FW. The differences were statistically significant in all three survey periods (p < 0.0001).

Tables 1, 2, and 3 present the percentage contributions of different tooth groups, in both the upper and lower arches, to the composition of the DMFT index according to exposure to FW in Brazilian state capitals in 2003, 2010, and 2023, together with the corresponding results of the hypothesis tests (p-values).

**Table 1:** Proportion of the contribution of dental groups to the DMFT index among 12-year-old children in Brazilian capitals with (n=3,271) and without (n=1,972) fluoridated water, in 2003.

Tooth group	Fluoridated water				p-value
	Yes		No		
	N	prop.	N	prop.	
CI	241	0.047	281	0.049	0.6242
LI	183	0.035	244	0.042	0.0602
C	19	0.004	33	0.006	0.1310
PM1	158	0.031	290	0.050	<0.0001 *
PM2	239	0.046	476	0.083	<0.0001 *
M1	3,635	0.705	3,664	0.636	<0.0001 *
M2	680	0.132	772	0.134	0.7566
TOTAL	5,155	1.000	5,760	1.000	-

Note 1: All groups correspond to permanent teeth, including maxillary and mandibular teeth from both hemiarches: central incisors (CI), lateral incisors (LI), canines (C), first premolars (PM1), second premolars (PM2), first molars (M1), and second molars (M2).

Note 2: *p-value corresponding to the z-test (α=0.05).

Source: Brazilian Ministry of Health. SB Brasil 2003 Project.

**Table 2:** Proportion of the contribution of dental groups to the DMFT index among 12-year-old children in Brazilian capitals with (n=3,407) and without (n=2,171) fluoridated water. Brazil, 2010.

Tooth group	Fluoridated water				p-value
	Yes		No		
	N	prop.	N	prop.	
CI	230	0.050	342	0.063	0.0052 *
LI	145	0.032	253	0.047	<0.0001 *
C	47	0.010	71	0.013	0.1676
PM1	180	0.039	327	0.060	<0.0001 *
PM2	295	0.064	448	0.083	<0.0001 *
M1	3,115	0.680	3,120	0.577	<0.0001 *
M2	572	0.125	848	0.157	<0.0001 *
TOTAL	4,584	1.000	5,409	1.000	-

Note 1: All groups correspond to permanent teeth, including maxillary and mandibular teeth from both hemiarches: central incisors (CI), lateral incisors (LI), canines (C), first premolars (PM1), second premolars (PM2), first molars (M1), and second molars (M2).

Note 2: *p-value corresponding to the z-test (α=0.05).

Source: Brazilian Ministry of Health. SB Brasil 2010 Project.

**Table 3:** Proportion of the contribution of dental groups to the DMFT index among 12-year-old children in Brazilian capitals with (n=2,336) and without (n=2,385) fluoridated water, in 2023.

Tooth group	Fluoridated water				p-value
	Yes		No		
	N	prop.	N	prop.	
CI	97	0.043	231	0.044	0.8494
LI	70	0.031	187	0.035	0.3844
C	43	0.019	133	0.025	0.1164
PM1	117	0.052	456	0.087	<0.0001 *
PM2	201	0.089	786	0.149	<0.0001 *
M1	1,422	0.633	2,594	0.492	<0.0001 *
M2	296	0.132	882	0.167	<0.0001 *
TOTAL	2,246	1.000	5,269	1.000	-

Note 1: All groups correspond to permanent teeth, including maxillary and mandibular teeth from both hemiarches: central incisors (CI), lateral incisors (LI), canines (C), first premolars (PM1), second premolars (PM2), first molars (M1), and second molars (M2).

Note 2: *p-value corresponding to the z-test (α=0.05).

Source: Brazilian Ministry of Health. SB Brasil 2023 Project.

**Figure 2:**
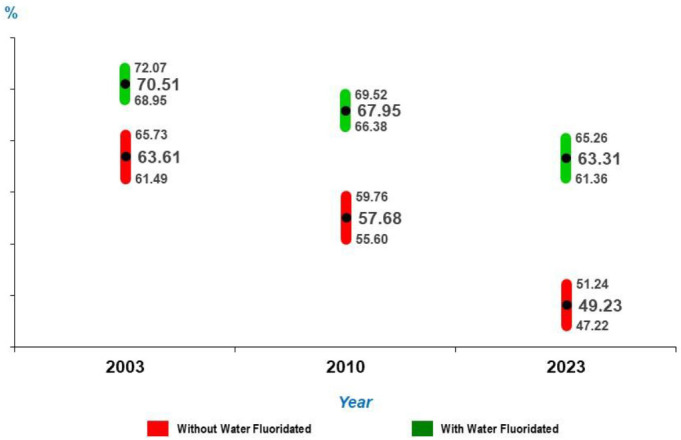
Percentage contribution (95%CI) of permanent first molars to the composition of the DMFT index at 12 years of age in Brazilian capitals with and without fluoridated public water supply, in 2003 (n=5,243), 2010 (n=5,578), and 2023 (n=4,721).

Source: Brazilian Ministry of Health. SB Brasil Projects 2003,2 010, and 2023.

Figure 2 specifically shows the percentages of M1 participation in the composition of the DMFT index, with their respective confidence intervals (α = 0.05). It can be observed that, in 2003, this participation was 70.51% where there was FW exposure and 63.61% where there was no FW. In 2010 and 2023, in turn, these participations were, respectively, 67.95% and 57.68% and 63.61% and 49.23.

## DISCUSSION

Across the three epidemiological surveys analyzed, DMFT index values were lower in capitals with FW, corroborating the scientific evidence regarding the efficacy and effectiveness of this public health intervention under the epidemiological conditions observed in Brazil^28^.

According to Rocca et al., the number of missing M1s and M1s requiring extraction among schoolchildren is 2 to 10 times lower in areas with FW^6^. In the present study, which focuses on the DMFT index rather than solely on decayed and extracted teeth — thereby including restored teeth — it was observed that the mean DMFT values in capitals with FW were 41% lower in 2003, 45% lower in 2010, and 57% lower in 2023. These findings corroborate evidence indicating that, within the Brazilian context, although community water fluoridation is not the only population-based preventive measure implemented, exposure to this intervention remains an effective strategy for reducing the burden of dental caries.

The main contribution of this study is the confirmation, previously unidentified in the empirical literature on the subject, that exposure to FW increases the proportional contribution of M1s to the DMFT index. Hypothesis testing indicates that this pattern was consistently observed across the three periods investigated, regardless of the significant differences in DMFT values over time, as shown in Figure 1.

Because incisors and canines do not present pits and fissures in their crowns, they are less susceptible to caries, consistent with the hierarchy of susceptibility according to tooth type and surface described as one of the universal patterns of disease distribution^1^. Another assumption is that molars and premolars, when exposed to FW, are protected to a degree comparable to other tooth types; however, due to the presence of pits and fissures, they would contribute proportionally more to the composition of the DMFT index. In the present study, however, this assumption, derived solely from clinical observation, could not be corroborated. Data presented in Tables 1, 2, and 3 show that, in all years analyzed, statistically significant differences indicate that M1s contribute proportionally more to the composition of the DMFT index under conditions of exposure to FW. This pattern was not observed for premolars and second molars, which, despite also presenting pits and fissures, contributed proportionally more to the composition of the index in the absence of FW. A hypothesis arising from this finding is that, beyond the presence of pits and fissures, the eruption period of M1s, occurring around 6 years of age, may render this tooth group proportionally less protected by exposure to FW.

On the other hand, although the results for 2003 and 2010 are inconclusive regarding the contributions of incisors and canines to the DMFT index, the findings for 2023 corroborate that the proportions contributed by these tooth groups to the index do not differ statistically significantly, regardless of exposure to FW^3^.

This represents the main finding of the present study, with potential implications for health planning, as it challenges prevailing assumptions and provides, in an original contribution to the international literature on the epidemiology of dental caries, empirical support for the hypothesis that, under conditions of exposure to FW, epidemiological surveillance strategies for dental caries should place increased emphasis on monitoring M1s.

### The preventiv e power of fluoridation

A statistically significant difference in mean DMFT scores was observed in 2003, 2010, and 2023 between Brazilian state capitals with and without FW. Although it is not possible to attribute the entire preventive effect underlying this phenomenon solely to FW, the contribution attributable to this variable should not be disregarded in analyses seeking to understand these findings more broadly within the framework of Evidence-Based Public Health^29^. It is also important to emphasize that the unit of analysis in this study was the sample element included in the surveys. Therefore, the results are not intended to estimate population parameters but rather to explore relative patterns (proportional contribution of M1s to the composition of the DMFT index).

In samples derived from population-based epidemiological surveys conducted in the analyzed state capitals, between 2003 and 2023, the DMFT index declined by approximately 39.2% in fluoridated capitals considered collectively and by 16.2% in non-fluoridated capitals in Brazil. This represents a substantial difference (58.7%), particularly in light of the socioeconomic conditions of the Brazilian population. From a public health perspective, this decline has been attributed to interventions with broad population reach, including exposure to fluoridated toothpaste; changes in oral health programs, which, during the transition to the 21st century, increasingly emphasized the importance of home-based oral health practices, such as tooth brushing and adherence to non-cariogenic diets; and, above all, the expansion of access to fluoridated public water supplies. Based on studies conducted in Brazil, the preventive fraction attributable to community water fluoridation in the specific Brazilian context has been estimated to range from 34% to 47% for the DMFT index^14,30^.

Analysis of the contribution of M1s to the composition of the DMFT index indicates that, even in contexts of low dental caries prevalence, exposure to FW contributes significantly to reducing the prevalence and severity of the disease^14,31^. Furthermore, the proportional contribution of M1s to the DMFT index continues to reproduce the pattern identified by Sheiham and Sabbah^1^. For this reason, the benefits associated with exposure to FW further reinforce the relevance of health surveillance strategies that recognize the importance of prioritizing M1s in the planning of public oral health actions. This is particularly relevant for active screening activities aimed at detecting incipient carious lesions, enabling enamel remineralization and control of cariogenic activity^32^, situations in which minimally invasive practices may be implemented^33^ and treatment of the disease may not require the use of rotary instruments^34^. In this regard, King et al. followed children aged 5 years (n=276) for 30 months, with examinations conducted twice yearly for caries detection, and schoolchildren aged 11 and 12 years (n=1,104), who were examined annually over a 36-month period. The authors observed that 50% of M1s had erupted by 6.4 years of age and that, 1 year after eruption, more than 10% of M1s and 45% of M2s already presented carious lesions. They concluded that, although caries progression was initially slower in M1s, the increase in disease occurrence within this dental group reached 18.8% between 7.5 and 8.5 years of age. By 15 years of age, the DMFT index reached 92% for M1s and 68% for M2s^35^.

### Implications of epidemiology for oral health surveillance actions

In Leeds, England, 128 children aged 11 years at baseline, during the late 1960s, had their M1s (n=512) monitored under epidemiological surveillance through annual examinations over a six-year period. At baseline, 12% of the participants were caries-free, 83% had between 1 and 3 carious or restored M1s, and 5% had all 4 M1s affected by caries or restorations. After 6 years of follow-up, 8% of the teeth that were initially caries-free had been extracted. Among the 83% of participants who initially presented with 1 to 3 carious or restored M1s, 19% lost these teeth during the follow-up period. In contrast, among the 5% with all 4 M1s affected by caries or restorations at baseline, 58% lost these teeth over the six years of monitoring^34^.

Within the context of Brazilian state capitals during the first decades of the 21st century, the focus of the present study, the main implication of the epidemiology of dental caries for oral health surveillance actions lies in the finding that, at the index age of 12 years, despite the expected decline in the prevalence and severity of caries, the hierarchical pattern of susceptibility according to tooth type persists. In this pattern, M1s remain the group most vulnerable to the disease and the group in which FW exerts a protective effect, albeit with comparatively lower preventive impact, as demonstrated in Tables 1, 2, and 3.

This finding does not corroborate the results reported by Singh et al., who observed that post-eruptive exposure of M1s to FW, in isolation, did not significantly reduce disease levels within this dental group^32^. Nevertheless, considering the aforementioned comparatively lower preventive effect, emphasis on the importance of actions that could be termed “epidemiological surveillance practices for the first permanent molar” during the post-eruptive maturation period of M1s is justified. From a broader public health programming perspective, this period may be defined as encompassing children aged 5 to 9 years.

It can be concluded that the benefits associated with exposure to FW further reinforce the relevance of health surveillance strategies that prioritize care directed toward M1s, suggesting that this focus should occupy a central role in such actions.

In the Brazilian context, sanitary conditions in state capitals exhibit distinct — and generally more favorable — patterns than those observed in other municipalities. This characteristic explains the decision, in the design of the present study, to use sample elements from state capitals, as data and information regarding public water supply are more readily available for these municipalities, thereby providing higher-quality data for analysis. However, this choice also represents a limitation of the study. Although data from reliable sources were used to characterize community water fluoridation, some degree of inaccuracy or incompleteness in these data cannot be ruled out. Furthermore, because studies involving FW are observational in nature rather than clinical trials, causal relationships cannot be inferred. This consideration should therefore be taken into account when interpreting the findings.

It is therefore important to emphasize the conclusion that exposure to FW does not diminish, but rather increases, the strategic relevance of M1s in the planning of collective oral health actions aimed at preventing dental caries and promoting its early treatment. Regardless of exposure to FW, the M1 group contributes substantially to the overall DMFT index value and should therefore receive priority attention within oral health surveillance practices. However, considering the evidence presented in this study demonstrating that exposure to FW significantly increases the proportional contribution of M1s to DMFT index values in samples from all three surveys (p < 0.0001 in all comparisons), attention directed toward this dental group is equally important in settings with and without FW. This constitutes the principal implication of the findings for health surveillance practices within the context of oral health actions.
