# Supplementary material for: Dental caries in permanent first molars: implications for health surveillance
Source: Rev Bras Epidemiol. 2026 Jul 31;29(Suppl 1):e260026supl1. doi: 10.1590/1980-549720260026.supl.1 (PMC13427311; doi:10.1590/1980-549720260026.supl.1)
Supplement: Translation

# Translation: Cárie em primeiros molares permanentes: implicações para a vigilância em saúde

**DOI:** 10.1590/1980-549720260026.supl.1.2

**Authors:** Paulo Capel Narvai, Antonio Carlos Frias, Carlos Cesar da Silva Soares, Jose Leopoldo Ferreira Antunes

**Affiliations:** I Universidade de São Paulo, Faculdade de Saúde Pública, Departamento de Política, Gestão e Saúde – São Paulo (SP), Brasil.; II Universidade de São Paulo, Faculdade de Odontologia, Departamento de Odontologia Social – São Paulo (SP), Brasil.; III Universidade de São Paulo., Faculdade de Saúde Pública, Departamento de Epidemiologia – São Paulo (SP), Brasil.

**Keywords:** Índice CPOD, Suscetibilidade à cárie dentária, Fluoretação da água, Programas preventivos de saúde, Vigilância da saúde

## Abstract

**Objetivo:**

Analisar a participação dos primeiros molares permanentes (M1) na composição do índice CPOD, com ou sem exposição à Fluoretação da Água (AF), e possíveis implicações para a vigilância em saúde.

**Métodos:**

Foram analisados dados secundários obtidos das amostras dos levantamentos nacionais de saúde bucal realizados pelo Ministério da Saúde em 2003, 2010 e 2023, na idade-índice de 12 anos, para as capitais estaduais brasileiras, com ou sem AF, calculando-se os valores médios do índice CPOD e as participações percentuais dos diferentes grupos dentários nesses valores.

**Resultados:**

As médias CPOD foram de 2,66, 2,49 e 2,21 para as capitais sem AF em 2003, 2010 e 2023, e de 1,58, 1,36 e 0,96, respectivamente, para as capitais com AF. Entre 2003 e 2023, o declínio das médias foi de 16,2% nas capitais sem AF e de 39,2% nas capitais com AF. Em 2003, a participação dos M1 no CPOD foi de 70,51% onde havia exposição à AF e de 63,61% onde não havia exposição. Em 2010 e em 2023, essas participações foram, respectivamente, de 67,95% e 57,68% e de 63,61% e 49,23%.

**Conclusão:**

A presença controlada de fluoreto na água contribuiu para o aumento na porcentagem da participação dos M1 na composição do índice CPOD, reduzindo-se essa proporção na ausência dessa medida preventiva, mas esse efeito não foi observado em pré-molares e segundos molares. Essa variação é relevante para as estratégias de vigilância em saúde bucal, notadamente as atividades de busca ativa de cárie incipiente e seu tratamento.

## INTRODUÇÃO

Os primeiros molares permanentes (M1) constituem o grupo de dentes mais suscetível à cárie. Por essa razão, a participação deste grupo no índice CPOD é bem conhecida dos epidemiologistas que se ocupam da distribuição dessa enfermidade em populações. De modo geral, essa proporção varia, em todas as idades, entre um e dois terços do valor do índice, independentemente da magnitude da doença. Os caninos compõem, por outro lado, o grupo menos atingido pela cárie^1^. No estudo pioneiro de Klein, Palmer e Knutson (1938), no qual o índice CPOD foi proposto, os caninos participaram com apenas 0,34% do valor total, no grupo de 6 a 15 anos (n = 4.416). Os M1, entretanto, contribuíram com 77,59% do valor, dos quais 51,43% corresponderam aos molares inferiores^2^.

Na idade-índice de 12 anos, as características da prevalência não impactam, significativamente, a mencionada proporção da participação dos M1 nos valores do índice CPOD. Admite-se que essa característica da distribuição da cárie decorre das características morfológicas das coroas dentárias dos diferentes grupamentos de dentes^3^.

Em Birmingham, no Reino Unido, abastecida com Água Fluoretada (AF) desde 1964, 40% das crianças de 11 anos apresentavam os M1 livres de cárie em 1980. Essa proporção foi comparada com crianças de mesma idade de Wolverhampton, não expostas à AF, entre as quais 78% apresentavam cárie em pelo menos 1 dos 4 M1. Observou-se que houve quase quatro vezes mais primeiros molares extraídos em Wolverhampton do que em Birmingham^4^. No Brasil, Lopes et al. avaliaram, em 1985, a prevalência de cárie em M1 em crianças que nasceram e sempre residiram em duas comunidades do Piauí: Teresina, com água de abastecimento público fluoretada (0,68 mgF/L), e Barras, sem AF. Embora não tenham analisado a participação dos M1 na composição do índice CPOD, os autores concluíram que a exposição à AF (Teresina) contribuiu para reduzir a magnitude com que a doença se manifestava, em todas as superfícies dentárias, inclusive dos M1, em comparação à observada na ausência da exposição (Barras)^5^.

Embora no contexto do planejamento e programação em saúde as características epidemiológicas dos M1, no conjunto dos grupos dentários, sejam de especial interesse para o planejamento e organização de ações de vigilância da saúde bucal, os conhecimentos consistentes sobre os M1 disponíveis na segunda metade do século XX – não apenas para a higidez, mas também para a boa qualidade da oclusão dentária – pareceram suficientes aos pesquisadores, pois o tema praticamente desapareceu da literatura relacionada com a prevenção da cárie. Nesse sentido, em 1979, Rocca et al. afirmaram que “são relativamente poucos os investigadores que têm se preocupado em avaliar os dados referentes, especificamente, aos primeiros molares, tendo em vista sua significação biológica e alta suscetibilidade ao ataque por lesões cariosas”^6^.

A exceção, no plano internacional, foi o estudo de Linkosalo^7^, que analisou a diferença na experiência de cárie em primeiros molares permanentes, calculando o índice CPOD especificamente para os M1, em dois grupos de crianças finlandesas nascidas em 1963, que viviam em cidades com e sem AF, concluindo que a exposição à AF, isoladamente, não é suficiente para constituir um efetivo regime preventivo. Entretanto, na primeira década do século XXI, Bachelor e Sheiham voltaram a dar atenção aos M1 e demonstraram que, comparativamente, as superfícies oclusais desses dentes são as mais suscetíveis à cárie. Assinalaram, também, que a ocorrência de cárie nas fóssulas e fissuras dos primeiros molares inferiores está fortemente associada ao risco de acometimento dos segundos molares e pré-molares^3^.

A prevalência, a incidência e as taxas de progressão de cárie, em dentes permanentes, seguem padrões reconhecidos como universais levados em conta por profissionais incumbidos do planejamento e programação em saúde e podem ser sumarizados como segue: a) níveis de cárie seguem linhas de tendência, o que implica reconhecer que, em determinada idade, podem assumir caráter preditivo em relação aos níveis observados em faixas etárias subsequentes; b) em termos populacionais, à medida que o CPOD médio aumenta, a porcentagem de indivíduos livres de cárie diminui e amplia-se a dispersão da doença na população; c) há uma relação matemática específica entre o CPOD médio (em que a unidade de medida é o dente) e o CPOS médio (em que a unidade de medida é a superfície dentária); d) há uma hierarquia de suscetibilidade à cárie por tipo de dente e superfície dentária, com padrões bem definidos de ocorrência; e) as alterações nas pontuações médias do CPOD para indivíduos e grupos não são lineares, mas escalonadas, com grupos de dentes e superfícies dentárias que apresentam “resistência” semelhante à cárie; f) à medida que o CPOD médio diminui, é maior a resistência, na fase de maturação pós-eruptiva, aos processos de desmineralização do esmalte dentário. Embora melhores condições populacionais de saúde bucal reduzam a progressão da doença, a hierarquia de suscetibilidade da cárie dentária mantém-se inalterada, independentemente da exposição ao flúor^1^.

Ainda que a segurança e a eficácia da fluoretação da água na prevalência e na magnitude da cárie sejam reconhecidas como evidência científica^8^, beneficiando, portanto, todos os grupos dentários, admite-se haver diferenças no modo como essa “força preventiva” se expressa nos distintos grupos de dentes.

Tendo em vista que a exposição à AF faz diminuir, de modo geral, a participação de incisivos e caninos na composição do índice CPOD, a implicação lógica é que em indivíduos expostos à AF ocorre aumento na proporção dos M1 nessa composição. Não obstante, ainda que não haja dúvida quanto à eficácia preventiva da AF sobre todos os grupos dentários, em razão da similaridade do modo de ação do fluoreto em todos os dentes, nos processos de des-remineralização do esmalte dentário, não está claro o grau da diferença entre as proporções de M1 no CPOD quando há e quando não há exposição à AF.

O pressuposto teórico deste artigo é de que há evidência científica da associação positiva entre exposição à água de abastecimento pública fluoretada e menor prevalência de cárie dentária. Contudo, no contexto do planejamento e programação em saúde bucal, tal associação pode levar à admissão de que, quando há exposição à AF e se admite sua efetividade preventiva, a proteção proporcionada pela medida seria proporcionalmente igual para todos os grupos dentários. Neste artigo, analisamos essa admissão, avaliando o grau da diferença entre as proporções de M1 na composição do índice CPOD, quando há e quando não há exposição à AF, para testar o quanto a exposição à AF modifica essa proporção, e as possíveis implicações para o planejamento de ações de vigilância em saúde bucal.

## MÉTODOS

Foram analisados dados secundários obtidos das amostras dos levantamentos nacionais de saúde bucal realizados pelo Ministério da Saúde, por meio do Projeto SB Brasil, em 2003 (n=5.243)^9^, 2010 (n=5.578)^10^ e 2023 (n=4.721)^11^, na idade-índice de 12 anos, para as capitais estaduais brasileiras, com ou sem exposição à AF^12^. Foram calculados os valores médios do índice CPOD e as participações percentuais dos diferentes tipos de dentes nesses valores. Foram considerados expostos à AF os elementos amostrais provenientes de capitais com pelo menos cinco anos continuados de fluoretação da água^13,14^. Os dados primários foram obtidos por inquéritos populacionais epidemiológicos que utilizaram a metodologia preconizada pela Organização Mundial da Saúde (OMS) sobre prevalência e magnitude da cárie dentária^15^.

A opção pela utilização de dados apenas das capitais estaduais, e não da população total do país (universo amostral disponível), decorre da precariedade das informações sobre a concentração de fluoretos em águas de abastecimento público no Brasil^16^ para o período deste estudo^17^. De acordo com o Sistema de Informação da Vigilância da Qualidade da Água para Consumo Humano (Sisagua), 31% dos municípios apresentam insuficiência de registros. Essa heterogeneidade comprometia a análise feita neste artigo. O Quadro 1 apresenta as listas das capitais com e sem AF em 2003, 2010 e 2023. A técnica de triangulação de fontes foi utilizada para confirmar as informações.

**Quadro 1:** Lista das capitais brasileiras com e sem água fluoretada em 2003 e 2010 e em 2023.

Ano	Água fluoretada	
	Sim (ano de início)	Não
2003 e 2010	Aracaju (1985) Belo Horizonte (1977) Brasília (1968) Campo Grande (1985) Curitiba (1958) Florianópolis (1982) Fortaleza (1989) Goiânia (1985) Porto Alegre (1975) Rio de Janeiro (1985) * Salvador (1996) São Paulo (1985) Teresina (1997) Vitória (1982)	Belém Boa Vista Cuiabá João Pessoa Macapá Maceió Manaus Natal Palmas Porto Velho Recife Rio Branco São Luís
2023	Às capitais com AF em 2003 e 2010, juntaram-se Cuiabá e Palmas em 2023. Palmas iniciou a fluoretação em 2016 e Cuiabá, em 2017.	Capitais sem AF em 2003 e 2010 mantiveram-se nessa categoria em 2023, com exceção de Cuiabá e Palmas.

AF: água fluoretada; *informação não pode ser confirmada por triangulação.

Fontes: Banco de dados do Projeto SB Brasil 2003; Banco de dados do Projeto SB Brasil 2010; Souza et al.^18^; Barros et al.^19^; Freire et al.^20^; Cardoso et al.^21^; Bleicher et al.^22^; Cesa et al.^23^; Silva et al.^24^; Gonçalves et al.^25^; Narvai et al.^12^; Peres et al.^26^.

A hipótese de igualdade nas proporções das participações dos diferentes tipos de dentes na composição do CPOD, com ou sem exposição à AF, foi testada para os anos de 2003, 2010 e 2023, empregando-se o teste z, com nível de significância de 5%^27^. Testes de hipótese foram realizados para cada grupo de dentes.

Para análise comparativa das médias do CPOD nas capitais com e sem AF, foi realizado o teste t, adotando-se o nível de significância de 5%^27^. É muito importante, contudo, ter em vista que o objetivo deste artigo não é dirigido à análise epidemiológica da cárie nas capitais com e sem AF. Por essa razão, os valores do CPOD são apresentados tal como foram divulgados após a conclusão dos inquéritos, pelo Ministério da Saúde. Assim, reiteramos que o objetivo deste artigo se refere, exclusivamente, aos padrões de distribuição da doença entre os diferentes grupamentos dentários, nas capitais com e sem AF, tal como se apresentam nas amostras dos inquéritos epidemiológicos que lhes deram origem.

Os referidos inquéritos populacionais contaram com planos amostrais complexos. Por essa razão, a análise empreendida neste artigo, tendo em vista o propósito do delineamento, está circunscrita às respectivas amostras. Essa menção aos planos amostrais complexos é necessária, pois este é um aspecto central da concepção e do método empregado neste estudo. As estatísticas não se referem, portanto, aos valores CPOD “cheios” das unidades de análise (as capitais) para a idade de 12 anos, mas apenas e tão somente aos M1, pois nosso objetivo, neste estudo, não é inferir parâmetros populacionais e analisar a prevalência da cárie nas capitais, estimada pelos valores “cheios” do índice CPOD. Ao nos ocuparmos apenas dos M1, para o propósito identificado no objetivo deste estudo, calculamos o CPOD “cheio” e apuramos as proporções com que os M1 participam dos respectivos valores.

### Declaração de Disponibilidade de Dados

Todo o conjunto de dados que dá suporte aos resultados deste estudo está disponível publicamente, mediante solicitação ao Ministério da Saúde, pelo e-mail: cosab@saude.gov.br.

## RESULTADOS

A Figura 1 mostra as médias e respectivos intervalos de confiança (95%) do índice CPOD em capitais com e sem AF. As diferenças são estatisticamente significativas nas três situações (*p* < 0,0001).

**Figura 1:** Valores médios do índice CPOD, aos 12 anos, para as capitais brasileiras com e sem água fluoretada, nos anos 2003 (n=5.243), 2010 (n=5.578) e 2023 (n=4.721).

Nota: o tamanho do círculo em torno da estimativa pontual da média indica a estimativa por intervalo de confiança (95%) do parâmetro popual cional.

Fonte: Ministério da Saúde. Projeto SB Brasil 2003, 2010 e 2023.

As Tabelas 1, 2 e 3 apresentam as porcentagens das participações dos dentes dos diferentes grupos, superiores e inferiores, na composição do índice CPOD, segundo a exposição à água fluoretada, em capitais brasileiras em 2003, 2010 e 2023, com os respectivos resultados dos testes de hipótese (p-valor).

**Tabela 1:** Proporção das participações dos grupos dentários no índice CPOD, em crianças de 12 anos, em capitais brasileiras com (n=3.271) e sem (n=1.972) água fluoretada, em 2003.

Dente	Água fluoretada				Valor p
	Sim		Não		
	N	prop.	N	prop.	
IC	241	0,047	281	0,049	0,6242
IL	183	0,035	244	0,042	0,0602
C	19	0,004	33	0,006	0,1310
PM1	158	0,031	290	0,050	<0,0001 *
PM2	239	0,046	476	0,083	<0,0001 *
M1	3.635	0,705	3.664	0,636	<0,0001 *
M2	680	0,132	772	0,134	0,7566
TOTAL	5.155	1,000	5.760	1,000	-

Nota 1: Todos os grupos correspondem a dentes permanentes, superiores e inferiores, das hemiarcadas inferiores e superiores: incisivos centrais (IC), incisivos laterais (IL), caninos (C), primeiros pré-molares (PM1), segundos pré-molares (PM2), primeiros molares (M1) e segundos molares (M2).

Nota 2: *valor de p correspondente ao teste z (α=0,05).

Fonte: Ministério da Saúde. Projeto SB Brasil 2003.

**Tabela 2:** Proporção das participações dos grupos dentários no índice CPOD, em crianças de 12 anos, em capitais brasileiras com (n=3.407) e sem (n=2.171) água fluoretada. Brasil, 2010.

Dente	Água fluoretada				Valor p
	Sim		Não		
	N	prop.	N	prop.	
IC	230	0,050	342	0,063	0,0052 *
IL	145	0,032	253	0,047	<0,0001 *
C	47	0,010	71	0,013	0,1676
PM1	180	0,039	327	0,060	<0,0001 *
PM2	295	0,064	448	0,083	<0,0001 *
M1	3.115	0,680	3.120	0,577	<0,0001 *
M2	572	0,125	848	0,157	<0,0001 *
TOTAL	4.584	1,000	5.409	1,000	-

Nota 1: Todos os grupos correspondem a dentes permanentes, superiores e inferiores, das hemiarcadas inferiores e superiores: incisivos centrais (IC), incisivos laterais (IL), caninos (C), primeiros pré-molares (PM1), segundos pré-molares (PM2), primeiros molares (M1) e segundos molares (M2).

Nota 2: *valor de p correspondente ao teste z (α=0,05).

Fonte: Ministério da Saúde. Projeto SB Brasil 2010.

**Tabela 3:** Proporção das participações dos grupos dentários no índice CPOD, em crianças de 12 anos, em capitais brasileiras com (n=2.336) e sem (n=2.385) água fluoretada, em 2023.

Dente	Água fluoretada				Valor p
	Sim		Não		
	N	prop.	N	prop.	
IC	97	0,043	231	0,044	0,8494
IL	70	0,031	187	0,035	0,3844
C	43	0,019	133	0,025	0,1164
PM1	117	0,052	456	0,087	<0,0001 *
PM2	201	0,089	786	0,149	<0,0001 *
M1	1.422	0,633	2.594	0,492	<0,0001 *
M2	296	0,132	882	0,167	<0,0001 *
TOTAL	2.246	1,000	5.269	1,000	-

Nota 1: Todos os grupos correspondem a dentes permanentes, superiores e inferiores, das hemiarcadas inferiores e superiores: incisivos centrais (IC), incisivos laterais (IL), caninos (C), primeiros pré-molares (PM1), segundos pré-molares (PM2), primeiros molares (M1) e segundos molares (M2).

Nota 2: *valor de p correspondente ao teste z (α=0,05).

Fonte: Ministério da Saúde. Projeto SB Brasil 2023.

A Figura 2 mostra, especificamente, as porcentagens das participações dos M1 na composição do índice CPOD, com os respectivos intervalos de confiança (α = 0,05). Observa-se que, em 2003, essa participação foi de 70,51% onde havia exposição AF e de 63,61% onde não havia AF. Em 2010 e em 2023, por sua vez, essas participações foram, respectivamente, de 67,95% e 57,68% e de 63,61% e 49,23.

**Figura 2:** Porcentagem da participação (IC95%) dos primeiros molares permanentes na composição do índice CPOD, aos 12 anos, em capitais brasileiras com e sem água de abastecimento público fluoretada, nos anos 2003 (n=5.243), 2010 (n=5.578) e 2023 (n=4.721).

Fonte: Ministério da Saúde. Projeto SB Brasil 2003, 2010 e 2023.

## DISCUSSÃO

Para os três inquéritos epidemiológicos analisados, os valores do CPOD são menores nas capitais onde há AF, corroborando a evidência científica sobre a eficácia e a efetividade, nas condições epidemiológicas do Brasil, dessa tecnologia de saúde pública^28^.

De acordo com Rocca et al., o número de M1 ausentes e com extração indicada em escolares é de 2 a 10 vezes menor em localidades com AF^6^. Neste estudo, cujo foco é o CPOD e não apenas dentes cariados e extraídos — incluindo, portanto, os dentes restaurados —, observa-se que o número médio de dentes CPOD em capitais com AF foi 41% menor em 2003, 45% em 2010 e 57% em 2023. Esses dados corroboram que, no contexto brasileiro, ainda que não seja *a* única medida preventiva massiva em implementação, a exposição à AF segue sendo uma medida efetiva.

A principal contribuição deste estudo é a confirmação, ainda não identificada na literatura empírica sobre o tema, de que a exposição à AF amplia a proporção com que os M1 participam da composição do índice CPOD. Os testes de hipótese indicam que essa característica se repete nos três períodos investigados, independentemente de os valores do CPOD diferirem significativamente na linha do tempo, conforme mostrado na Figura 1.

Por não apresentarem fóssulas e fissuras nas coroas, os dentes incisivos e caninos apresentam menor suscetibilidade à cárie, em consonância com a hierarquia de suscetibilidade por tipo dentário e superfície descrita como um dos padrões universais da distribuição dessa doença^1^. Outra suposição é que molares e pré-molares são igualmente protegidos em comparação com outros tipos de dentes, quando expostos à AF, mas, por terem fóssulas e fissuras, participariam em maior proporção do índice CPOD. Neste estudo, porém, essa suposição, derivada apenas da observação clínica, não pode ser corroborada. Observa-se nos dados das Tabelas 1, 2 e 3 que, em todos os anos analisados, as diferenças estatisticamente significativas indicam que os M1 participam proporcionalmente mais da composição do índice CPOD quando há exposição à AF. Entretanto, isso não ocorre com os pré-molares e os segundos molares, os quais, ainda que apresentem fóssulas e fissuras, participam proporcionalmente mais da composição do índice quando não há exposição à AF. A hipótese a ser extraída dessa constatação é que, mais importante do que apresentar fóssulas e fissuras, o período da erupção dentária dos M1, por volta dos 6 anos, pode tornar este grupo, proporcionalmente, menos protegido pela exposição à AF.

Por outro lado, embora os resultados para 2003 e 2010 sejam ambíguos quanto às participações de incisivos e caninos na composição do índice CPOD, os resultados para 2023 corroboram que as proporções com que esses grupos compõem o índice não diferem estatisticamente, de modo significativo, havendo ou não exposição à AF^3^.

Este é o principal resultado deste estudo, com potencial para contribuir para o planejamento em saúde, pois refuta o senso comum e proporciona, de modo original na literatura internacional sobre a epidemiologia da cárie, bases empíricas para a hipótese de que, quando há exposição à AF, as estratégias de vigilância epidemiológica da cárie dentária devem redobrar a atenção sobre os M1.

## A força preventiva da fluoretação

Houve diferença estatisticamente significativa nas médias do CPOD observadas em 2003, 2010 e 2023 entre as capitais brasileiras que contam com AF e as que não dispõem dessa medida preventiva. Ainda que não seja possível atribuir à AF toda a força preventiva implicada nesse fenômeno, o efeito atribuível a essa variável não deveria ser desconsiderado em análises que busquem compreender, amplamente, à luz do referencial da Saúde Pública Baseada em Evidências^29^, o significado mais profundo desses fatos. Importa assinalar, ainda, que a unidade de análise neste artigo é o elemento amostral integrante dos inquéritos, motivo pelo qual os resultados não pretendem estimar parâmetros populacionais, mas explorar padrões relativos (proporções dos M1 no CPOD).

Nas amostras dos inquéritos epidemiológicos populacionais provenientes das capitais analisadas, no período compreendido entre 2003 e 2023, o CPOD registrou um declínio da ordem de 39,2% nas capitais fluoretadas consideradas conjuntamente, e de 16,2% nas capitais não fluoretadas no Brasil. Trata-se de uma diferença (58,7%) não trivial, considerando as condições socioeconômicas da população brasileira. Para especialistas em saúde pública, na base desse declínio estão ações de amplo alcance populacional, como a exposição a cremes dentais fluoretados; mudanças nos programas de saúde bucal, que, na transição para o século XXI, passaram a enfatizar ainda mais a importância de cuidados domésticos com a saúde bucal, como a escovação dental e dietas não cariogênicas; e, sobretudo, a ampliação do acesso à água de abastecimento público fluoretada. Com base em estudos realizados no Brasil, a fração preventiva que pode ser atribuída à fluoretação, no específico contexto brasileiro, variou de 34% a 47% no índice CPOD^14,30^.

A análise das participações de M1 na composição do CPOD indica que, mesmo em contextos de baixa prevalência de cárie dentária, a exposição à AF contribui, significativamente, para a redução da prevalência e da magnitude da doença^14,31^. Além disso, a participação dos M1 no percentual do índice CPOD segue reproduzindo o padrão identificado por Sheiham e Sabbah^1^. Por essa razão, o benefício proporcionado pela exposição à AF torna ainda mais relevantes as estratégias de vigilância em saúde que reconheçam a importância da atenção que deve ser dada aos M1, ao planejar ações de saúde pública na área de saúde bucal, notadamente as atividades de busca ativa de cárie incipiente para remineralização do esmalte e controle da atividade cariogênica^32^, situações em que se efetivam práticas minimamente invasivas^33^ e nas quais a terapia para a doença dispensa o emprego de instrumentos rotatórios^34^. A esse respeito, King et al. acompanharam crianças de 5 anos (n=276) durante 30 meses, examinando-as duas vezes por ano para detecção de cárie, e escolares de 11 e 12 anos (n=1.104), examinando-as anualmente, durante 36 meses. Observaram que 50% dos M1 erupcionaram aos 6,4 anos e que, 1 ano após a erupção, mais de 10% dos M1 e 45% dos M2 já estavam cariados. Concluíram que, embora o desenvolvimento de cáries tenha sido inicialmente mais lento nos M1, o incremento nesse grupo dentário aumentou para 18,8% entre as idades de 7,5 e 8,5 anos. Aos 15 anos, o CPOD era de 92% para os M1 e de 68% para os M2^35^.

## Implicações da epidemiologia para as ações de vigilância em saúde bucal

Em Leeds, Inglaterra, 128 crianças inicialmente com 11 anos, no final dos anos 1960, tiveram seus M1 (n=512) sob vigilância epidemiológica, com exames anuais, durante 6 anos. Inicialmente, 12% dos examinados estavam livres de cárie, 83% apresentavam de 1 a 3 M1 cariados ou restaurados, e 5% apresentavam os 4 M1 cariados ou restaurados. Após 6 anos, 8% dos dentes originalmente livres de cárie haviam sido extraídos. Dos 83% que apresentavam de 1 a 3 M1 cariados ou restaurados, 19% haviam perdido esses dentes; e dentre os 5% que apresentavam os 4 M1 cariados ou restaurados, 58% os perderam ao longo dos 6 anos que durou o monitoramento^34^.

No contexto das capitais brasileiras, nas primeiras décadas do século XXI, objeto deste estudo, a principal implicação da epidemiologia da cárie dentária para as ações de vigilância em saúde bucal é a constatação de que, na idade-índice de 12 anos, não obstante o esperado declínio observado na prevalência e magnitude da cárie, reproduz-se o padrão hierárquico de suscetibilidade por tipo dentário – com os M1 representando o grupo mais vulnerável à doença e nos quais AF produz efeito protetor, porém relativamente com menor “força preventiva”, conforme as Tabelas 1, 2 e 3.

Tal resultado não corrobora a constatação de Singh et al. de que a exposição pós-eruptiva dos M1 à AF, isoladamente, não reduziu significativamente os níveis da doença nesse grupo dentário^32^. Não obstante, tendo em vista a mencionada menor força preventiva relativa, justifica-se ressaltar a importância de ações que se poderiam denominar de “práticas de vigilância epidemiológica do primeiro molar permanente” no período de maturação pós-eruptiva dos M1, definido mais amplamente, em termos de programação de saúde pública, como abrangendo o grupo etário de 5 a 9 anos.

Pode-se concluir que o benefício da exposição à AF torna ainda mais relevantes as estratégias de vigilância em saúde que atribuem importância à atenção a se dispensar aos M1, que deve estar no centro dessas ações.

No contexto brasileiro, a situação sanitária das capitais apresenta padrões distintos – e, de modo geral, melhores – do que os demais municípios. Decorre disso a opção, no delineamento deste estudo, pelos elementos amostrais relativos às capitais, pois para esses municípios estão disponíveis dados e informações sobre *a* água de abastecimento público que conferem melhor qualidade aos dados que utilizamos. Entretanto, tal escolha implica uma limitação do presente estudo. Ainda que tenhamos utilizado dados provenientes de fontes confiáveis para informações sobre a fluoretação das águas, pode haver, em algum grau, deficiência em alguns desses dados. Decerto que, em delineamentos que envolvam AF, não há que falar em relações causais, pois não se está perante algum tipo de ensaio clínico. Assim, o leitor deve ter isso em conta.

Cabe, portanto, enfatizar a conclusão de que a exposição à AF não diminui, mas, ao contrário, faz aumentar a relevância estratégica dos primeiros molares permanentes no planejamento de ações coletivas em saúde bucal orientadas à prevenção da cárie e ao seu tratamento imediato. Independentemente da exposição à AF, o grupamento M1 contribui majoritariamente para o valor do CPOD cheio e deve receber atenção prioritária nas práticas de vigilância da saúde bucal. No entanto, considerando que, conforme demonstramos, a exposição à AF altera, significativamente, para mais a participação do M1 nos valores do índice CPOD nas amostras dos 3 inquéritos (p < 0,0001 nas 3 comparações), a atenção a ser dada a esse grupamento dentário é tão importante onde há quanto também onde não há AF. É essa a implicação, mencionada, para as práticas de vigilância em saúde no contexto das ações de saúde bucal.

## CÓDIGO DE ÉTICA:

Os projetos de pesquisa dos inquéritos cujos dados secundários foram utilizados neste estudo foram submetidos e aprovados, oportunamente, pela Comissão Nacional de Ética em Pesquisa. Tendo em vista as características deste estudo, não houve necessidade de sua apreciação, específica, no âmbito do sistema CEP/CONEP.
